# The association between suicidal risk and ADHD comorbid with mood disorders in adults

**DOI:** 10.1192/j.eurpsy.2026.10440

**Published:** 2026-07-31

**Authors:** C. Hallal, A. Kassab, E. El-Dirani, Y. Toum, L. Abi Khalil, F. Zahran, M. Louak, R. Bou Khalil

**Affiliations:** 1 Saint-Joseph University; 2 Psychiatry, Hôtel-Dieu de France, Beirut, Lebanon

## Abstract

**Introduction:**

Mood disorders, particularly bipolar disorder and major depressive disorder, are common and often debilitating psychiatric conditions. These disorders frequently co-occur with other mental illnesses, especially attention-deficit/hyperactivity disorder (ADHD), which may significantly impact their clinical trajectory and overall burden. While prior research has linked comorbid ADHD and mood disorders to an elevated risk of suicidality, such studies have generally failed to account for potential moderating variables, including symptom severity, comorbid psychopathologies, and ADHD subtype heterogeneity.

**Objectives:**

This study aims to investigate the relationship between suicidality and ADHD, in its distinct subtypes, within the context of mood disorders, taking into consideration contributing factors that may modulate disease severity and influence suicidal risk, but also analyzing the differential impact of ADHD subtypes: inattentiveness subtype, impulsivity subtype and low self-esteem/negative mood subtype.

**Methods:**

Data were collected through a structured online questionnaire completed by patients hospitalized for mood disorders in the psychiatry department of Hôtel-Dieu de France between 2017 and 2025. It covered sociodemographics, ADHD screening (Wender Utah Rating Scale WURS-25 and Adult ADHD Self-Report Scale ASRS), past and current suicidal risk, illness course since discharge, comorbid substance use disorders, and ongoing psychiatric treatment.

**Results:**

The study analyzed 49 responses. Suicidality was present in 73.5% of participants, with 65.3% reporting prior behavior and 18.4% prospective risk. Overall ADHD was not significantly linked to suicidality (p > .05). However, when examining ADHD subtypes, logistic regression showed age was protective (OR = 0.91, p = .011), inattentiveness reduced risk (OR = 0.77, p = .040), and impulsivity increased risk (OR = 1.14, p = .042). Post-hospitalization substance use was also associated with suicidality (χ²(1) = 4.54, p = .033). After mood adjustment, inattentiveness lost significance. Predictive models explained up to 34.9% of variance, highlighting the roles of age, ADHD profiles, mood, and substance use in suicidal vulnerability.

**Conclusions:**

Suicidal risk in this population appears multifactorial, influenced by age, ADHD subtypes, substance use and mood. Identifying protective and risk factors is essential for early intervention. Future research should further clarify these interactions to improve prevention strategies.

**Keywords:**

Bipolar disorder, major depressive disorder, mood disorders, ADHD, suicidal risk, suicide, adults.

**Disclosure of Interest:**

None Declared

